# Linguistic validation and cultural adaptation of an English version of the Evaluation of Daily Activity Questionnaire in rheumatoid arthritis

**DOI:** 10.1186/s12955-014-0143-y

**Published:** 2014-09-20

**Authors:** Alison Hammond, Sarah Tyson, Yeliz Prior, Ruth Hawkins, Alan Tennant, Ulla Nordenskiold, Ingrid Thyberg, Gunnel Sandqvist, Ragnhild Cederlund

**Affiliations:** Centre for Health Sciences Research (OT), University of Salford, Frederick Road, M6 6PU Salford, UK; Stroke & Vascular Research Centre, School of Nursing, Midwifery & Social Work, University of Manchester, Manchester, UK; Centre for Health Sciences Research (OT), University of Salford, Salford, UK; Derby branch National Rheumatoid Arthritis Society (patient research partner), Derby, UK; ICF Research Branch, Swiss Paraplegic Research (previously: Academic Department of Rehabilitation Medicine, Section of Musculoskeletal Disease, Leeds Institute of Molecular Medicine, Faculty of Medicine and Health, The University of Leeds), Nottwil, Switzerland; Institute of Neuroscience and Physiology, Rehabilitation Medicine, Sahlgrenska Academy, Göteborg University, Göteborg, Sweden; Department of Clinical and Experimental Medicine, Linköping University, Linköping, Sweden; Department of Rheumatology, Lund University, Lund, Sweden; Department of Health Sciences, Lund University, Lund, Sweden

**Keywords:** Patient reported outcome measures, Rheumatoid arthritis, Daily activities, Occupational therapy

## Abstract

**Background:**

To linguistically validate and culturally adapt the Evaluation of Daily Activity Questionnaire (EDAQ) for use in rheumatoid arthritis (RA) from Swedish to British English. The EDAQ is a patient reported outcome measure of daily activity ability. It includes 11 activity domains (Eating and Drinking; Personal Care; Dressing; Bathing; Cooking; Moving Indoors; House Cleaning; Laundry; Moving and Transfers; Communication; Moving Outdoors) and was developed for use in rheumatoid arthritis (RA).

**Methods:**

The EDAQ was translated from Swedish to English using standard methods. Activity diaries, cognitive debriefing interviews and focus groups were completed with people with RA to: generate new culturally applicable items; identify important items in the Swedish version to retain in the English version; and develop the English EDAQ based on their views of content and layout. Content validity was established by linking the EDAQ to the International Classification of Functioning RA Core Set.

**Results:**

The English EDAQ translation was harmonized with the Swedish version to ensure equivalence of meaning. Sixty-one people with RA participated. 156 activities were identified from 31 activity diaries and included in a draft English EDAQ. Following interviews (n = 20) and four focus groups, 138 activities were retained and three additional domains added (Gardening/Household Maintenance; Caring; and Leisure/Social Activities). Most ICF RA Core Set activities are in the EDAQ.

**Conclusions:**

The English EDAQ is a detailed self-report measure of ability in RA with good content validity.

**Electronic supplementary material:**

The online version of this article (doi:10.1186/s12955-014-0143-y) contains supplementary material, which is available to authorized users.

## Background

People with arthritis easily identify major difficulties with every-day activity, but specific ones can be harder to articulate. People are busy getting on “living their lives” rather than closely monitoring their problems. Yet difficulty with many simple things (e.g. using a computer, turning taps, gripping) limit ability to work, do housework, cook, manage self-care and leisure, causing frustration, pain and fatigue. Helping people with arthritis to identify (and manage) such problems is a key aspect of occupational therapy (OT) but this is hampered by a lack of effective assessment tools.

Most standardised assessments have relatively few physical function items (for example, the Health Assessment Questionnaire (HAQ) [1] and Arthritis Impact Measurement Scales 2 (AIMS2) [2]). Consequently, they are seldom used in OT practice as they lack the detail to identify problems. In contrast, the Evaluation of Daily Activity Questionnaire (EDAQ) is a self-report assessment which facilitates detailed assessment, treatment planning and outcome measurement in rheumatoid arthritis (RA) [3,4]. It was developed, and is used in Sweden in both OT practice and research [5–9]. It has three parts:

1. details of the patients’ disease duration, patient education programme attendance and 10 visual analogue scales (VAS) measuring symptom severity, mood and life satisfaction;
2. 11 domains assessing 102 daily activities including Eating, Dressing, Personal Care, Bathing, Cooking, Cleaning the House, Laundry, Communication, Moving Indoors, Transfers and Moving Outdoors. Each domain is assessed in two sections: (A) evaluates activities without using ergonomic methods or help; and (B) with ergonomic methods (e.g. joint protection, activity pacing, assistive technology). The activities with which patients are least and most satisfied with are also recorded; and
3. a checklist regarding the use of assistive devices (40 items).

The EDAQ is completed by people at home, allowing time to reflect, before or between OT appointments [10].

Although published in English, the Swedish EDAQ has limitations. It was not professionally translated meaning some activities are unclear (e.g. “managing potato water”). Some commonly problematic activities for people with RA in the United Kingdom (UK) are not included, e.g. using a kettle, turning taps. Furthermore, the EDAQ was developed in the 1990’s and some activities are now infrequent (e.g. writing a postcard, taking bread from the oven) and others, not included, are commonplace (e.g. using a computer). Also only women with RA helped develop its content. Thus the EDAQ needs to be revised to include activities that all people with RA in the UK consider problematic, ensuring it reflects users’ perspectives [11,12].

Consequently, the aims of this study were to: linguistically validate and culturally adapt the EDAQ into British English; and revise it to include daily activities people with RA consider problematic, important and culturally relevant in the UK, establishing its content validity. We also investigated validity by: matching the EDAQ with the daily activities Rheumatology occupational therapists (OTs) commonly assess, as well as their opinions of the EDAQ; and systematically linking the EDAQ with the International Classification of Function, Disability and Health (ICF) [13] and the ICF Core Set for RA [14].

## Methods

Four phases were conducted: linguistic validation into British English using forward and backward translation; cultural adaptation and item generation using activity diaries, cognitive debriefing interviews and focus groups; content analysis of OT activity assessments; and linking the EDAQ with the ICF and ICF Core Set for RA.

Ethical approval was obtained from Oldham Local Research Ethics Committee (09/H1011/25) and the University of Salford’s Ethics Committee. The study was carried out in accordance with the Declaration of Helsinki.

### Linguistic validation

Recommended procedures were followed [15,16] as follows:

1. The meaning of EDAQ content was identified at a ‘conceptual definition’ meeting with the Swedish EDAQ research team (UN, IT, GS, RC).
2. Independent professional forward translation from Swedish to British English by two translators (one informed and one uninformed about the study purpose; both bilingual but native Swedish speakers).
3. Synthesis and refinement of the translations by members of the UK research team (AH, ST, RH)
4. Independent backward translation from British English into Swedish by two different translators (one informed and one uninformed; both bilingual but native British English speakers).
5. Synthesis by the Swedish research team.
6. Harmonization between the UK and Swedish research teams to ensure equivalence of meaning.

### Cultural adaptation and item generation

#### Participants

Adults with RA attending five rheumatology out-patient departments were invited to participate if they were able to read, write and understand English. Patients were excluded if they had other medical condition(s) causing difficulties with daily activities. Participants were purposefully sampled to ensure a range of age, disease duration, disability, employment and living/family status. A minimum of 30 people were therefore required. All participants were provided with study information verbally and in writing and provided written consent.

#### Procedures

To check applicability of Swedish EDAQ items and generate any new items for the English EDAQ, people with RA completed activity diaries [5,17,18], recording problematic activities for seven days, including weekend days. Following development of a draft English EDAQ, including revised instructions and a Part 2 completed example, semi-structured cognitive debriefing interviews were conducted with people with RA who participated in the activity diary phase. Participants were timed completing the draft English EDAQ and asked about: ease of completion; length; clarity of instructions; layout; how important they considered it to include each item on a scale of 1 (not at all) to 5 (extremely); whether any important activities were omitted; and if the EDAQ would give an OT sufficient insight into their daily activity problems. Categorical responses were provided and any comments recorded verbatim. Focus groups with people with RA were then conducted, asking for consensus within groups about the wording of the introduction and instructions, layout and which items to add, delete or merge. All had completed the EDAQ beforehand.

#### Data analysis

Diaries were content analysed by two reviewers independently to identify activities reported as causing difficulties, their frequency and then agreeing analyses [19]. These activities were cross-matched with items in Part 2 of the EDAQ. New activities identified as problematic were added to a draft English language EDAQ. From the cognitive debriefing interviews, medians (IQRs) for importance responses for each item were calculated, with items scoring 3 or less (ie not at all to moderately important) considered for deletion. Importance ratings were reviewed by two researchers, and new activities and potential items for deletion or merging identified. The research team finalised the English EDAQ using these patient-generated decisions.

### Content of occupational therapists’ daily activity assessments and their views of the EDAQ

Members of the North West College of OTs’ Specialist Section-Rheumatology (NWCOTSS-R: n = 23) were asked for their standardized and non-standardized daily activity assessments. These were content analyzed and cross-matched to identify to what extent these overlapped with the draft EDAQ Part 2. The OTs were also asked to rate importance of including items. OT ratings were compared with participants with RA’s ratings, using Mann – Whitney U tests, to identify if any differences in opinion about item inclusion. A focus group with Rheumatology OTs was conducted to: identify if any common problems treated were omitted in Part 2; ensure Part 3 included the most commonly recommended assistive devices; and discuss the EDAQ’s potential use in practice. Recommendations were considered when finalizing the EDAQ.

### ICF linking

To evaluate content validity of the EDAQ, items were systematically linked by two researchers (AH, YP) to the International Classification of Functioning, Disability and Health (ICF) [13] and ICF Core Set for RA [14] using the ICF linking rules [20,21].

## Results

### Linguistic validation

Several changes were made following forward translation. For example, the mood VAS in Part 1 translated to “high and low spirited.” These terms are not in everyday use in British English and we decided “happy and unhappy” would better capture mood. Sixteen Swedish activities were modified because they were rarely performed in the UK and were replaced by more generic activities, for example: “take bread out of the oven” to “take things out of the oven”; “drain potato water” to “drain water from a pan (e.g. pasta, vegetables)”; “write a postcard” to “write”.

### Cultural adaptation and item generation

Activity diary respondents’ characteristics are shown in Table 1. Forty-two people agreed to participate. There were no significant differences in age, gender, disease duration or severity, activity ability, employment or children living at home status between responders (n = 31) and non-responders (n = 11). Of the 102 activities in Part 2 of the Swedish EDAQ: 84 were identified in participants’ diaries; and 18 were not. Fifty-four new items were generated: 26 were incorporated into the original Part 2 domains of the Swedish EDAQ and a further 28 were fitted into three new domains; Gardening and House Maintenance, Caring, and Leisure and Social Activities. Thus, Part 2 of the draft EDAQ was extended to 14 domains, including 156 activities (Additional file 1).

**Table 1:** **Participant characteristics in the item generation and cognitive debriefing interview phases**

	**Item generation (activity diary) phase (Responders: n = 31)**	**Item generation (activity diary) phase (Non-responders: n = 11)**	**Interview phase: (n = 20)**
Average age (years)	60.29 (SD 15.43)	54.36 (SD 14.32)	61.55 (SD 17.1)
Gender M:F (n)	7:24	2:9	4:16
Disease duration (years)	13.14 (SD 12.17)	12.68 (SD 11.84)	16.43 (SD 14.14)
Health assessment questionnaire (0–3)	0.64 (SD 0.47)	0.89 (SD 0.47)	0.87 (SD 0.78)
Perceived disease severity (0–10 scale)	4.31 (SD 2.25)	4.43 (SD 2.14)	4.76 (SD 2.20).
Employed (n)	7	6	4
Children under 18 years living at home (n)	8	6	4

Cognitive debriefing interview participants’ characteristics are shown in Table 1 (n = 20). The draft EDAQ took 48 (SD 19; range 22 to 84) minutes to complete. Those taking longer had breaks to avoid fatigue and hand pain. Participants rated all Part 1 rating scales as very or extremely important (Table 2). For items in Part 2, the median (IQR) scores for the importance of activities are shown in Additional file 1. Participants rated 145/156 items as either very or extremely important. All assistive devices (Part 3) were rated as very or extremely important, primarily because it helped participants be aware of what was available. Only a few additional items were suggested by participants, most of which were already in the EDAQ. Work was recommended to be added (n = 3) and a question therefore included in Part 1, to prompt OTs to use a work assessment if necessary.

**Table 2:** **Median (IQR) importance ratings and ICF codes for EDAQ Part 1 scales**

**Part one: numeric rating scales**	**RA participants’ ratings (n = 20)**	**OT participants’ ratings (n = 11)**	**p**	**ICF code**	**ICF category**
Disease activity	4 (4–5)	4 (4–5)	0.85	nd-ph	Not definable- physical health
Mood	4 (4–5)	4 (4–5)	0.67	b152	Emotional functions
Pain when resting	5 (4.25-5)	4 (4–5)	0.12	b280	Sensation of pain
Pain when moving	5 (4.25-5)	5 (4–5)	0.92	b280	Sensation of pain
Stiffness	5 (4–5)	4 (4–5)	0.28	b7800	Sensation of muscle stiffness
Limitations in joint movement	5 (4.25-5)	5 (4–5)	0.61	b710	Mobility of joint functions
Fatigue	5 (4.25-5)	5 (4–5)	0.92	b130	Energy and drive functions
Worry	4 (4–5)	4 (4–5)	0.56	b152	Emotional functions
Sleep problems	4 (4–5)	5 (4–5)	0.67	b134	Sleep problems
Satisfaction with life	4 (4–5)	4 (4–5)	0.61	nd-gh	Not definable- general health
Additional questions: work (paid, unpaid) or education					d850 Remunerative employment; d855 Non-remunerative employment; d825 Vocational training; d830 Higher education; d839 Education, other
Patient education programme attendance					d570 Looking after one’s health

Fifteen considered the assessment easy to complete; two partially (having to re-read instructions); and three not easy. Two were unclear how to complete Part 2B and one did not like thinking about difficulties. Most (n = 15) considered the EDAQ had “about the right amount” of questions. However, five said it had too many questions and 10 considered it took too long. Recommendations to shorten it included: combining activities where possible (for example, “Slicing food (eg bread and cheese)” rather than separate items); deleting uncommon items (eg going out onto a balcony, opening a lift door); omitting the sections in Part 2 about the least and most satisfactory items, other problems and solutions (n = 6); and making Part 3 optional (n = 5). Some considered the Caring domain less important (n = 7).

Eighteen considered the EDAQ Part 2 Sections A and B layout easy to follow but five commented the instructions and example needed careful reading first. Half (n = 12) considered the instructions adequate, but seven wanted more (one did not reply). Recommendations were to: add a “not applicable” column in Section A; emphasizing that section B is not completed if activities are not applicable or not difficult; clarifying how to answer if the person has help; and including a wider range of answers in the example. These changes were made.

Four focus groups were conducted with four to six participants with RA. The introduction content was agreed. The consensus was to retain all 10 scales in Part 1 but use horizontal 0-10 numeric rating scales (NRS) rather than 100mm VAS as NRS were considered easier to complete, and the wording of scale anchors was agreed. A consensus regarding the layout was finalized. For Part 2, five activities were deleted and 25 combined into 13 activities (see Additional file 1). All 14 domains were kept. Although Caring was a possible exclusion, many looked after grandchildren. Gardening and Leisure were considered especially important to retain as “*there is more to life than just doing everyday activities*.” The revised Part 2 thus included 138 activities in 14 domains (Additional file 1). The “most and least satisfactory activities” sections were removed and Part 3 made optional, as device use is noted in Section B of Part 2.

The final EDAQ normally takes 25 to 35 minutes to complete. Almost all (n = 19) considered the EDAQ would help OTs gain adequate insight into their difficulties, (one could not answer as she had never seen OTs).

### Content of occupational therapists’ daily activity assessments and their views of the EDAQ

Twenty NWCOTSS-R members responded, submitting assessments from 17 departments. All used their own department-devised checklists. Standardized assessments were occasionally used by six departments. These were the HAQ (n = 5); Disability Arm Shoulder Hand scale [22]: n = 3); AIMS2 (n = 2). Checklists included 33 (IQR 23–45; range 5 to 55) daily activities. All items on the checklists and assessments were matched to the items in the draft EDAQ and corresponded to 91 activities (Additional file 1). Eleven OTs rated the importance of draft EDAQ activities; 83/156 were thought to be very or extremely important to include (Additional file 1). OTs rated 83 (53%) activities of similar importance as participants with RA, 9 (6%) as more important and 64 (41%) less important. In the focus group (n = 7), no additional activities were recommended and the assistive device list was modified to include devices commonly recommended in the UK and ensure terminology was correct. Initially, OTs were concerned by the EDAQ’s length because it would take too long during an appointment and some patients would be unable or uninterested in doing it. We explained the EDAQ is completed at home in the person’s own time and most participants with RA considered it acceptable and relevant. This allayed their concerns and they reflected:

*“It might save time …if they do it in their own time, it is taking out the time of having to actually go through it all with them. You've got your talking points there straight away, which helps focus a lot more [OT2].”*

### ICF linking

The EDAQ has good content validity as all but three ICF RA Core Set Activities and Participation items (32 categories) are included. Part 2 items relate to 6/9 ICF Activities and Participation chapters (see Additional file 1).

## Discussion

In this study, we linguistically validated and culturally adapted a British English version of the Evaluation of Daily Activity Questionnaire, based on what participants with RA in the UK considered the most important content. We established the EDAQ’s content validity for the first time. Future research will establish the psychometric properties of the EDAQ in RA and other musculoskeletal conditions.

A strength of this study is that it meets requirements for patient reported outcome measures, as it was developed from the people with RA’s perspectives [11,12]. The OT participants considered fewer activities very or extremely important to include than people with RA. Patients’ perspectives can differ from health professionals, which is why patients’ views should be incorporated when developing patient reported outcomes [23,24]. As one EDAQ respondent commented:

*“Little things to help can make life better…. It’s stupid little things; the EDAQ shows those small things”.*

Additionally, the EDAQ measures activity both without and with ergonomic solutions, allowing both therapist and patient to evaluate their impact. Initially, we anticipated fewer activities would be included but were surprised that most participants with RA considered the longer draft EDAQ necessary to identify their problems sufficiently. They were however concerned how long it took, so we followed their advice to delete or merge activities (by 10%) and made Part 3 optional, reducing completion time by a third. Although it takes about 35 minutes, people normally do it at their leisure over several days at home, allowing time for reflection.

The EDAQ has good content validity based on the ICF Core Set for RA activities as almost all are included and it has more activities related to Domestic Life than most other measures [25,26]. The limitations of this study are that personal and intimate relationships (ICF Chapter 7) were not identified as problematic in the diary, interview or focus group stages, although people with RA can find these difficult. These are not included in the Swedish EDAQ and our instructions may have been too focused on everyday activities for people to consider relationships as appropriate. Including more people with RA in the EDAQ’s development or changing the instructions given could have identified further problematic activities. However, the EDAQ cannot include everything; it is not intended to replace a clinical interview and this may be a more appropriate setting for such topics. Additionally, during forward and backward translation, we used professional translators and thus did not include a translator with a clinical background. However, the research team included experienced Rheumatology occupational therapists (AH, UN, IT, GS, RC), the Swedish research team (UN, IT, GS, RC) are bilingual and the meaning of EDAQ content was discussed extensively, during both the two day conceptual definition meeting and later teleconferences following the translations, to clarify meaning.

Developing the English EDAQ for the UK demonstrated why assessments from other countries must be culturally adapted as well as translated. We identified activities commonly problematic in the UK, such as using kettles and teapots, turning taps and flushing toilets, which were not in the Swedish EDAQ. The conceptual definition meeting identified why. The Swedes are a nation of ‘real coffee’ drinkers using coffee machines, rather than kettles, whereas two-thirds of Britons drink tea daily [27]. Sweden adopted principles of universal design in the 1970’s, thus most Swedish taps and toilets are easy to use. In the UK, this is still not the case so difficulties persist. Similarly, the EDAQ requires cultural adaptation for other countries as activities may be problematic in the UK but not elsewhere. For example, UK electric plugs are hard to push and pull but much smaller and easier to use in the USA.

## Conclusions

The English EDAQ is a detailed measure of daily activity ability, reflecting what people with RA consider is most important to include, with good content validity. As a comprehensive measure of activities commonly found difficult by people with RA in the UK, the EDAQ has the potential to be both a useful clinical tool and an outcome measure for research. Completing it at home saves time in OT appointments and it could facilitate in-depth discussion between client and therapist as they jointly seek solutions and treatments to help. Further testing is needed to establish reliability and validity.

## Additional file

Additional file 1:
**Frequency of Swedish EDAQ and UK activities recorded in activity diaries and OT assessments; participants’ and OTs’ activity importance ratings (median, IQR) for English EDAQ inclusion; differences between participants’ with RA and OTs’ ratings; and ICF codes.**
