# Supplementary material for: Linguistic validation and cultural adaptation of an English version of the Evaluation of Daily Activity Questionnaire in rheumatoid arthritis
Source: Health Qual Life Outcomes. 2014 Sep 20;12:143. doi: 10.1186/s12955-014-0143-y (PMC4209054; doi:10.1186/s12955-014-0143-y)
Supplement: Additional file 1: — Frequency of Swedish EDAQ and UK activities recorded in activity diaries and OT assessments; participants’ and OTs’ activity importance ratings (median, IQR) for English EDAQ inclusion; differences between participants’ with RA and OTs’ ratings; and ICF codes. [file 12955_2014_143_MOESM1_ESM.doc]

**Additional file 1.** Frequency of Swedish EDAQ and UK activities recorded in activity diaries and OT assessments; participants’ and OTs’ activity importance ratings (median, IQR) for English EDAQ inclusion; differences between participants’ with RA and OTs’ ratings; and ICF codes

| Domain and item | No. Activity Diary citations (n = 31) | No. OT Assessment citations (n = 20) | Median (IQR) participants’ importance ratings (n = 20) | Median (IQR) OTs’ importance ratings (n = 11) | p | ICF Code/ ICF Category |
| --- | --- | --- | --- | --- | --- | --- |
| 1. EATING & DRINKING |  |  |  |  |  | d550; d560 |
| 1. Lift a glass | 1 | 8 | 4 (3–5) | 3 (3–4) | 0.50 | d560 Drinking |
| 2. Lift a cup /mug | 2 | 10 | 4 (3–5) | 5 (5–5) | 0.01* | d560 Drinking |
| 3. Use a knife and fork | 5 | 13 | 4 (4–5) | 5 (5–5) | 0.01* | d550 Eating |
| 4. Slice food (eg bread^1^ cheese^1^ ) | - | - |  |  |  | d440 Fine hand use |
| Slice bread^1^ | 1 | 2 | 3 (2–5) | 2 (2–3) | 0.08 |  |
| Slice cheese ^1^ | 0 | 0 | 3 (2–4.25) | 2 (2–2) | 0.01* |  |
| 5. Get milk out of fridge | 1 | 0 | 3 (3–4) | 3 (3–4) | 0.50 | d445 Hand and arm use (includes d4450pull/ d4451 push fridge door; d4452 reach for milk); d4401 grasping (milk) |
| 6. Open a milk carton /plastic bottle ^1^ and pour out | - | - |  |  |  | d440 Fine hand use; d4453 turning hands/arms |
| Open a plastic milk carton/ plastic bottle ^1^ | 4 | 6 | 4 (3.25-5) | 4 (4–5) | 0.98 |  |
| Pour out milk ^1^ | 2 | 0 | 4 (3–4) | 3 (3–5) | 0.89 |  |
| 7. Open a bottle top (eg lager) | 7 | 3 | 4 (3–5) | 3 (3–5) | 0.42 | d440 Fine hand use |
| 8. Open a screw top jar or bottle^1^ | - | - |  |  |  | d4453 Turn/ twist hands or arms |
| Open a screw top jar^1^ | 10 | 13 | 4.5 (4–5) | 4 (4–5) | 0.79 |  |
| Open a screw top bottle ^1^ | 7 | 4 | 4.5 (4–5) | 4 (3–5) | 0.47 |  |
| 9. Open a tin or a ring-pull can | 6 | 9 | 4 (4–5) | 4 (4–5) | 0.87 | d4453 Turn/ twist hands or arms; d4402 Manipulating |
| 10. Open a packet/pouch | 3 | 0 | 4 (2.75-5) | 4 (3–4) | 0.83 | d4402 Manipulating |
| 2. IN THE BATHROOM & PERSONAL CARE |  |  |  |  |  | d510,d520, d530 |
| Get to the toilet | 0 | 0 | 4 (3.25-5) | 5 (5–5) | 0.01* |  |
| 1. Get on and off the toilet | 1 | 15 | 4 (4–5) | 5 (5–5) | 0.003* | d420 Transferring oneself |
| 2. Wipe yourself with toilet paper /clean self below ^1^ | - | - |  |  |  | d530 Toileting |
| Wipe yourself with toilet paper | 1 | 11 | 4.5 (4–5) | 5 (5–5) | 0.02* |  |
| Wash your private parts after going to toilet ^1^ | 1 | 8 | 5 (3.25-5) | 5 (5–5) | 0.23 |  |
| 3. Use suppositories/tampons | 0 | 0 | 4 (3–4.25) | 3 (2–4) | 0.08 | d5302 Menstrual care |
| 4. Flush the toilet | 2 | 1 | 4 (3.75-5) | 4 (4–5) | 0.94 | d445 Hand and arm use |
| 5. Arrange your clothes after going to toilet | 0 | 0 | 4 (3–5) | 5 (4–5) | 0.18 | d5408 Dressing, other unspecified |
| 6. Wash your hands | 1 | 2 | 5 (4–5) | 5 (4–5) | 0.94 | d5100 Washing body parts |
| 7. Brush and comb your hair | 1 | 6 | 4 (4–4) | 4 (4–5) | 0.20 | d5202 Caring for hair |
| 8. Brush your teeth | 1 | 11 | 4 (4–5) | 4 (4–5) | 0.84 | d5201 Caring for teeth |
| 9. Use a tube of toothpaste | 1 | 3 | 4 (3–4) | 4 (3–5) | 0.97 | d440 Fine hand use; d5201 Caring for teeth |
| 10. Open a medicine bottle/ blister pack | 4 | 4 | 5 (4–5) | 5 (4–5) | 0.59 | d4453 Turn/ twist hands or arms |
|  |  |  |  |  |  | d440 Fine hand use |
| 11. Do your make up or shave | 1 | 12 | 4 (3–4) | 4 (4–4) | 0.56 | d5200 Caring for skin |
| 12. Put on jewelry/watch | 0 | 0 | 3 (3–4) | 3 (2–3) | 0.04* | d440 Fine hand use |
| 3. GETTING DRESSED & UNDRESSED |  |  |  |  |  | d540 |
| 1. Put on / take off a coat | 0 | 3 | 4 (3.25-4.75) | 3 (3–4) | 0.15 | d5400/ Putting on clothes |
|  |  |  |  |  |  | d5401 Taking off clothes |
| 2. Pull clothes over your head | 1 | 8 | 4 (3–4.75) | 4 (4–5) | 0.13 | d5400 Putting on clothes |
| 3. Put on front-opening clothes | 0 | 5 | 4 (3–5) | 4 (3–5) | 0.78 | d5400 Putting on clothes |
| 4. Do up/undo buttons | 4 | 13 | 4 (4–5) | 4 (3–5) | 0.40 | d4402 Manipulating |
| 5. Pull clothes over your feet | 3 | 7 | 4 (4–5) | 4 (4–5) | 0.56 | d5400 Putting on clothes |
| 6. Do up /undo zips | 4 | 11 | 4 (3.25-5) | 4 (3–4) | 0.41 | d4402 Manipulating |
| 7. Put on tights/socks | 3 | 9 | 4 (4–4.75) | 4 (3–5) | 0.54 | d5400 Putting on clothes |
| 8. Take shoes/boots on and off ^1^ | - | - |  |  |  | d5402 Putting on footwear |
|  |  |  |  |  |  | d5403 Taking off footwear |
| Take shoes on and off^1^ | 6 | 8 | 4 (4–5) | 4 (3–5) | 0.85 |  |
| Take boots on and off ^1^ | 1 | 0 | 4 (4–5) | 3 (2–3) | 0.001* |  |
| 9. Tie shoelaces | 1 | 0 | 5 (4–5) | 3 (2–4) | 0.01* | d4402 Manipulating |
| 10. Put on/take off gloves | 0 | 9 | 3 (3–5) | 3 (2–3) | 0.02* | d5400/ Putting on clothes |
|  |  |  |  |  |  | d5401 Taking off clothes |
| 11. Fasten clothes at the back | 2 | 4 | 4 (4–5) | 3 (2–4) | 0.01* | d4459 Hand and arm use, unspecified; d4402 Manipulating |
| 4. BATHING & SHOWERING |  |  |  |  |  | d510,d520 |
| Get to and from the bathroom | 0 | 0 | 5 (4–5) | 4 (3–5) | 0.73 |  |
| 1. Get in and out of the bath | 13 | 15 | 5 (4–5) | 4 (3–5) | 0.03* | d420 Transferring oneself |
| 2. Shower whilst standing | 8 | 13 | 5 (4–5) | 5 (3–5) | 0.58 | d5101 Washing whole body; |
|  |  |  |  |  |  | d4154 Maintain a standing position |
| 3. Use shower controls /bath temperature mixers | 1 | 0 | 5 (4–5) | 4 (4–5) | 0.89 | d4402 Manipulating |
| 4. Turn taps | 7 | 10 | 4 (4–5) | 5 (4–5) | 0.49 | d4401 Grasping |
| 5. Wash your back and neck | 5 | 9 | 5 (4–5) | 4 (3–5) | 0.09 | d5100 Washing body parts |
| 6. Dry your back and neck | 3 | 8 | 5 (4–5) | 4 (4–5) | 0.12 | d5102 Drying oneself |
| 7. Wash and dry your feet | 0 | 6 | 5 (4–5) | 4 (4–5) | 0.05* | d5100 Washing body parts |
|  |  |  |  |  |  | d5102 Drying oneself |
| 8. Wash your hair | 6 | 13 | 4 (4–5) | 4 (4–5) | 0.38 | d5100 Washing body parts |
| 9. Style / blow-dry your hair ^1^ | - |  | 4 (3–4) | 4 (3–4) | 0.43 | d5202 Caring for hair |
| Style your hair ^1^ | 1 | 5 | 4 (3–4) | 4 (3–4) | 0.43 |  |
| Blow dry your hair ^1^ | 4 | 0 | 4 (3–4) | 4 (3–4) | 0.38 |  |
| 10. Cut/file your finger nails | 3 | 2 | 4 (4–5) | 3 (3–4) | 0.01* | d5203 Caring for fingernails |
| 11. Take care of your feet | 3 | 3 | 5 (4–5) | 4 (3–5) | 0.02* | d5204 Caring for toenails, d520 Caring for body parts |
| 5.COOKING |  |  |  |  |  | d630 |
| Move around in the kitchen | 0 | 1 | 4 (3–5) | 4 (4–5) | 0.60 |  |
| 1. Stand while working in the kitchen | 6 | 0 | 4 (3–5) | 4 (3–5) | 0.52 | d4154 Maintaining a standing position |
| 2. Set the table/ carry plates, cups etc. | 5 | 4 | 4 (3–5) | 3 (3–4) | 0.31 | d430 Lifting and carrying objects; (d4300 Lifting; d4301 Carrying in the hands; d4305 Putting down objects) |
| 3. Peel and chop vegetables | 14 | 12 | 4 (4–5) | 4 (3–4) | 0.26 | d6300 Preparing meals |
| 4. Carry a full pan to the cooker^1^ | - | - |  |  |  | d6300 Preparing meals; d4301 Carry in hands |
| Put a full saucepan on the cooker ^1^ | 12 | 8 | 5 (4–5) | 3 (3–4) | 0.001* |  |
| Carry a frying pan to/from the cooker with one hand ^1^ | 0 | 0 | 5 (4–5) | 3 (2–3) | 0.01* |  |
| 5. Drain water from a saucepan (e.g. vegetables, pasta) | 1 | 4 | 4 (4–5) | 4 (3–5) | 0.15 | d4300 Lifting; d4453 Turning or twisting in hands and arms; d6300 Preparing meals |
| 6. Remove heavy items (e.g. bag of sugar) from top cupboards | 1 | 7 | 4 (3.75-5) | 3 (2–3) | 0.01* | d4308 Lifting and carrying, other specified |
| 7. Baking (eg. cakes, bread, pastry) | 4 | 0 | 4 (3–4) | 2 (2–3) | 0.001* | d6308 Preparing meals, other specified |
| 8. Take things in/out of oven | 7 | 1 | 5 (4–5) | 4 (3–4) | 0.03* | d4300 Lifting; d4301 Carrying in hands. (if low oven, d4105 Bending) |
| 9. Wash up | 7 | 4 | 4 (3–5) | 3 (3–4) | 0.28 | d6401 Cleaning cooking area and utensils |
| 10. Put crockery/pans etc. into kitchen cupboards | 3 | 0 | 4 (3–4) | 3 (2–3) | 0.01* | d4300 Lifting : d4301 Carrying in the hands; d4305 Putting down items |
| 11. Use a kettle (e.g. fill, pour) | 10 | 11 | 5 (4–5) | 5 (5–5) | 0.31 | d4300 Lifting : d4301 Carrying in the hands; d4305 Putting down items |
| 12. Turn cooker knobs | 3 | 4 | 5 (3.5-5) | 4 (4–5) | 0.94 | d4453 Turning or twisting the hands or arms |
| 13. Open fridge door | 2 | 0 | 4 (3–5) | 4 (3–5) | 0.69 | d4450 Pulling |
| 14. Prepare and cook a snack and/or a meal | 9 | 10 | 4 (4–5) | 5 (4–5) | 0.23 | d6300/1 Preparing meals – simple / complex |
| 6. MOVING INDOORS |  |  |  |  |  | d410- d440 |
| 1. Walk indoors (e.g. get to toilet/ bathroom; round kitchen) | 7 | 11 | 4 (4–5) | 5 (5–5) | 0.05* | d4600 Moving around within the home |
| 2. Open the front or back door | 3 | 5 | 5 (4–5) | 5 (4–5) | 0.52 | d4453 Turning or twisting hands or arms; d4450 Pulling |
| 3. Lock and unlock doors (Open a Yale lock) | 3 | 13 | 5 (4–5) | 5 (4–5) | 0.98 | d4453 Turning or twisting hands or arms |
| 4. Get to the front door in time to answer | 0 | 0 | 4 (4–5) | 4 (3–5) | 0.85 | d4600 Moving around within the home |
| Open door to the balcony | 0 | 0 | 3.5 (2.75 – 5) | 1 (1–2) | 0.40 |  |
| Go outside on the balcony | 0 | 0 | 3 (1.75-4.25) | 3 (3–4) | 0.01* |  |
| 5. Get to the phone in time to answer | 0 | 0 | 4 (4–5) | 3 (3–4) | 0.01* | d4600 Moving around within the home |
| 6. Stand for longer periods | 9 | 1 | 5 (4–5) | 3 (2–4) | 0.001* | d4154 Maintaining a standing position |
| 7. Get up and down steps/ stairs | 7 | 6 | 5 (4.5-5) | 5 (4–5) | 0.64 | d469 Walking and moving, other specified |
| 8. Bend to floor/pick up items | 10 | 7 | 5 (4–5) | 3 (3–4) | 0.01* | d4105 Bending |
| 9. Reach up | 10 | 6 | 4 (4–5) | 3 (3–4) | 0.06 | d4452 Reaching |
| 10. Kneel | 8 | 0 | 5 (3–5) | 2 (1–3) | 0.001* | d4102 Kneeling; d4152 Maintaining a kneeling position |
| 11. Carry heavy items around the house | 4 | 0 | 5 (4–5) | 3 (2–3) | 0.001* | d4308 Lifting and carrying objects, other specified |
| 12. Manage heating (e.g. controls, woodburner, multifuel stove, open fire) | 1 | 7 | 4 (3–5) | 4 (4–5) | 0.94 | d449 Carrying, moving and handling objects, other specified and unspecified |
| 7. CLEANING THE HOUSE |  |  |  |  |  | d640 |
| 1. Make the bed | 14 | 5 | 4 (4–5) | 3 (3–4) | 0.002* | d6408 Doing housework, other specified |
| 2. Dust and wipe surfaces | 6 | 4 | 4 (3–4) | 3 (3–4) | 0.06 | d6408 Doing housework: other specified |
| 3. Sweep/mop floor | - | - | - | - | - | d6408 Doing housework: other specified |
| Sweep floor ^1^ | 1 | 0 | 4 (3–4) | 3 (2–4) | 0.01* |  |
| Mop floor ^1^ | 2 | 0 | 4 (3–4) | 3 (2–4) | 0.01* |  |
| 4. Wring out a cloth | 2 | 5 | 4 (3–4) | 4 (3–4) | 0.21 | d4453 Turning or twisting hands or arms |
| 5. Vacuum clean | 14 | 10 | 4 (4–5) | 3 (3–4) | 0.002* | d6402 Cleaning living area |
| 6. Open a window | 4 | 0 | 4 (3–5) | 3 (3–4) | 0.06 | d4458 Hand and arm use, other specified |
| 7. Clean windows | 3 | 1 | 4 (3–4.5) | 2 (2–3) | 0.002* | d6408 Doing housework: other specified |
| 8. Clean the bath | 3 | 0 | 4 (4–5) | 3 (2–3) | 0.001* | d6408 Doing housework: other specified |
| 9. Heavy housework (e.g. move furniture, take down curtains) | 10 | 11 | 4.5 (3–5) | 2 (2–2) | 0.002* | d6408 Doing housework: other specified |
| 8. LAUNDRY & CLOTHES CARE |  |  |  |  |  | d640 |
| 1. Do the hand washing | 1 | 2 | 3 (3–4) | 3 (2–3) | 0.004* | d6400 Washing and drying clothes and garments |
| 2. Use a washing machine (e.g. load and unload) | 3 | 3 | 4 (3–5) | 4 (3–5) | 0.77 | d6403- Using household appliances d6400 Washing and drying clothes and garments |
| 3. Hang out washing | 6 | 1 | 4 (3–5) | 3 (3–4) | 0.05* | d6400 Washing and drying clothes and garments |
| 4. Plug in and pull out a plug | 2 | 6 | 4 (4–5) | 4 (3–5) | 0.31 | d4401 Grasping; d4402 Manipulating |
| 5. Put up an ironing board | 1 | 0 | 4 (4–5) | 3 (2–3) | 0.002* | d4458 Hand and arm, other specified |
| 6. Iron | 10 | 7 | 4 (3–5) | 3 (3–4) | 0.12 | d6408 Doing housework: other specified |
| 7. Do small repairs e.g. hemming, buttons | 0 | 0 | 4 (3–5) | 2 (1.75-2) | 0.001* | d6500 Making and repairing clothes |
| 8. Cut cloth and/ or use scissors | 2 | 4 | 4 (3–5) | 2 (1–3) | 0.01* | d4402 Manipulating |
| 9. Pick up pins/needles | 0 | 0 | 4 (3–5) | 2 (1–2) | 0.001* | d4400 Picking up |
| 9.MOVING & TRANSFERS |  |  |  |  |  | d410,d420 |
| 1. Get into and out of bed^1^ | - | - |  |  |  | d420- Transferring oneself; d4106- Shifting the body's centre of gravity- d4100 Lying down |
| Get into bed ^1^ | 2 | 15 | 4 (4–5) | 5 (5–5) | 0.02* |  |
| Get out of bed ^1^ | 10 | 15 | 4 (4–5) | 5 (4–5) | 0.05* |  |
| 2. Turn-over and sit up in bed | 3 | 4 | 5 (4–5) | 5 (5–5) | 0.03* | d4108 Changing basic body position, other specified |
| 3. Stand up from a chair without armrests | 7 | 15 | 5 (4–5) | 4 (3–5) | 0.03* | d4104 Standing |
| 4. Pull up bedclothes/duvet | 3 | 0 | 4 (4–5) | 4 (4–5) | 0.96 | d4401Grasping; d5700 Ensuring one's physical comfort |
| 5. Getting a comfortable sleeping position | 12 | 0 | 5 (4–5) | 5 (4–5) | 0.80 | d4150 Maintaining a lying position; d5700- Ensuring one's physical comfort |
| 6. Sit for longer periods (e.g. in a car, train) | 6 | 0 | 4 (4–5) | 3 (2–3) | 0.001* | d4153 Maintaining a sitting position |
| 10.COMMUNICATION |  |  |  |  |  | d340,d360 |
| 1. Use a phone or mobile (e.g. call or text) | 3 | 6 | 5 (4–5) | 5 (4–5) | 0.45 | d3600 Using telecommunication devices |
| 2. Hold a book | 3 | 1 | 4 (3–5) | 3 (3–4) | 0.26 | d4401 Grasping |
| 3. Write | 7 | 13 | 5 (4–5) | 4 (4–4) | 0.29 | d345 Writing messages |
| 4. Handle money and credit/debit cards (eg use cash machine, pay by card) | 2 | 5 | 4 (3–5) | 4 (4–4) | 0.27 | d4408 Fine hand use, other specified; d860 Basic economic transactions; |
| 5. Use a computer and a mouse | 5 | 1 | 4 (4–5) | 3 (3–4) | 0.03* | d3601 Using writing machines |
| 6. Use remote controls (e.g. TV) | 2 | 0 | 4 (3.5-5) | 4 (3–4) | 0.11 | d4408 Fine hand use, other specified; |
| 11.MOVING OUTDOORS & SHOPPING |  |  |  |  |  | d4 |
| 1. Walk on level ground | 16 | 13 | 5 (4–5) | 5 (4–5) | 0.73 | d450 Walking |
| 2. Go for a long walk (e.g. a mile) | 16 | 3 | 4 (4–5) | 3 (2–3) | 0.002* | d4501 Walking long distances |
| 3. Go up stairs without a handrail | 9 | 13 | 5 (4–5) | 3 (3–4) | 0.001* | d4558 Moving around, other specified |
| 4. Travel by public transport | 3 | 10 | 5 (4–5) | 3 (3–4) | 0.005* | d4702 Using public motorized transportation |
| 5. Get in and out of a car | 7 | 6 | 5 (4–5) | 4 (3–5) | 0.01* | d4701 Using private motorized transportation; d4208 Transferring oneself, other specified |
| 6. Drive a car (e.g. hold steering wheel, turn car key, change gear) | 12 | 13 | 5 (4–5) | 4 (4–4) | 0.02* | d4751 Driving motorized vehicles |
| 7. Fill the car with petrol | 1 | 0 | 5 (4–5) | 3 (2–4) | 0.001* | d6503 Maintaining vehicles |
| 8. Open a heavy (eg shop) door^1^ | 4 | 8 | 4 (4–5) | 2 (2–4) | 0.02* | d4450 Pulling; d4451 Pushing; d4453 Turning or twisting the hands or arms |
| Open a lift door | 0 | 0 | 3 (2–5) | 2 (2–2) | 0.02* |  |
| 9. Walk around the shops | 7 | 0 | 4 (4–5) | 3 (3–4) | 0.02* | d4601 Moving around within buildings other than the home; d4602 Moving around outside the home and other buildings |
| 10. Carry shopping | 15 | 5 | 5 (4–5) | 3 (3–4) | 0.003* | d4301 Carrying in the hands |
| 11. Do the weekly shopping | 15 | 13 | 5 (4–5) | 4 (3–4) | 0.01* | d6200 Shopping |
| 12. Hold a walking stick | 1 | 2 | 4 (4–5) | 4 (4–5) | 0.46 | d4401 Grasping; d465 Moving around using equipment |
| 13. Use a mobility scooter | 1 | 0 | 5 (4.25-5) | 3 (3–4) | 0.001* | d4701 Using private motorized transportation; d465 Moving around using equipment |
| 12. GARDENING & HOUSE MAINTENANCE |  |  |  |  |  |  |
| 1. Change a light bulb | 1 | 4 | 4 (4–5) | 2 (2–3) | 0.001* | d4452 Reaching; d4453 Turning or twisting the hands or arms; d6501 Maintaining dwelling and furnishings; |
| 2. Light gardening (e.g. weed, prune, plant) |  |  |  |  |  | d6505 Taking care of plants inside and outside; d4101 Squatting; d4105 Bending |
| Weed ^1^ | 1 | 0 | 3 (3–4) | 2 (2–3) | 0.003* |  |
| Prune ^1^ | 4 | 0 | 3 (3–4) | 2 (2–3) | 0.004* |  |
| Plant out ^1^ | 3 | 0 | 4 (3–5) | 2 (2–3) | 0.002* |  |
| 3. Heavy gardening (eg dig, mow) |  |  |  |  |  | d6505 Taking care of plants, indoors and outdoors; d430 Lifting and carrying objects |
| Mow ^2^ | 1 | 0 | 3 (3–4.5) | 2 (2–3) | 0.004* |  |
| Heavy gardening ^2^ | 7 | 9 | 4.5 (3–5) | 2 (2–2) | 0.001* |  |
| 4. Climb ladders | 2 | 0 | 4 (2.5-5) | 2 (1–2) | 0.002* | d4551 Climbing |
| 5. Clean the car (inside and out) | 1 | 0 | 3.5 (3–4.75) | 2 (1–2) | 0.001* | d6503 Maintaining vehicles |
| 6. Do household repairs | 1 | 0 | 4 (3–5) | 2 (2–3) | 0.003* | d6501 Maintaining dwelling and furnishings |
| 7. Car maintenance (eg oil, water) | 2 | 0 | 4 (3–5) | 1 (1–3) | 0.003* | d6503 Maintaining vehicles |
| 13 CARING |  |  |  |  |  | d660 |
| 1. Feed a child, prepare bottles | 2 | 0 | 5 (3–5) | 5 (5–5) | 0.40 | d6604 Assisting others in nutrition |
| 2. Bathe a child/ change nappies | 2 | 0 | 5 (4–5) | 5 (5–5) | 0.23 | d6600 Assisting others in self-care |
| 3. Dress a child | 2 | 0 | 4 (4–5) | 5 (5–5) | 0.15 | d6600 Assisting others in self-care |
| 4. Do a child’s hair | 1 | 0 | 4 (3–5) | 4 (3–5) | 0.83 | d6600 Assisting others in self-care |
| 5. Use children’s equipment (e.g. high chair, push chair, car seat) | 3 | 0 | 4 (4–5) | 4 (4–5) | 0.82 | d6601 Assisting others in movement |
| 6. Put a child in/ out of high chair, push chair, high seat | 5 | 0 | 5 (4–5) | 5 (4–5) | 0.88 | d6601 Assisting others in movement |
| 7. Lift and carry a child | 2 | 0 | 4 (3–5) | 4 (4–5) | 0.68 | d6601 Assisting others in movement |
| 8. Play with children | 2 | 0 | 4 (3–5) | 4 (4–5) | 0.68 | d9200 Play |
| 9. Care for others (e.g. elderly relatives) | 1 | 0 | 4.5 (4–5) | 4 (3–5) | 0.41 | d669 Caring for household objects; d6601Assisting others, other specified and unspecified |
| 14.LEISURE & SOCIAL ACTIVITIES |  |  |  |  |  | d910, d920, d930 |
| 1. Crafts (e.g. knitting, crochet, sewing, embroidery, model making) | 7 | 1 | 4 (3–5) | 3 (3–4) | 0.07 | d9203 Crafts |
| 2. Do-It-Yourself (e.g. using tools, painting and decorating) | 1 | 1 | 4 (4–5) | 3 (2–3) | 0.001* | d9204 Hobbies; |
| 3. Visit friends/ socializing (eg pub, theatre, cinema) | 4 | 2 | 4 (4–5) | 4 (4–5) | 0.54 | d9205 Socializing |
| 4. Attend community / religious groups or classes | 1 | 2 | 4 (3.5-5) | 4 (3–4) | 0.17 | d910 Community life; d9300 organized religion |
| 5. Physical activities (e.g. dance, active sports, swimming, cycling) | 3 | 4 | 5 (4–5) | 4 (3–4) | 0.003* | d9208 Recreation and leisure, other specified; d4552 running; d9201 sports; d4554 swimming; d4700 using human-powered vehicles |
| 6. Quiet recreation (e.g. painting, cards) | 1 | 4 | 4 (4–5) | 3 (3–4) | 0.003* | d9208 Recreation and leisure, other specified; |
| 7. Performing arts (e.g. music, choir, dramatics) | 1 | 0 | 4 (3.5-5) | 3 (2–4) | 0.002* | d9202 Arts and culture |
| 8. Pet care (e.g. feed, groom) | 1 | 0 | 4 (3–5) | 3 (3–4) | 0.06 | d6505 Taking care of animals |
| 9. Take dog for a walk (e.g. hold leash) | 7 | 0 | 4 (3–5) | 3 (3–4) | 0.10 | d6505 Taking care of animals; d4401 Grasping |

Key:

Bold items = new English EDAQ and partially altered Swedish items; Italicised items: Swedish EDAQ activities deleted from the English EDAQ.

^1^ = Swedish EDAQ activities combined into one item; * significant difference between RA participants’ and OTs’ ratings (p ≤ 0.05).
